# Towards the development of a national patient transfer document between residential and acute care—A pilot study

**DOI:** 10.1111/opn.12374

**Published:** 2021-03-24

**Authors:** Dympna Tuohy, Anne Fahy, Jane O'Doherty, Pauline Meskell, Pauline O'Reilly, Brid O'Brien, Jill Murphy, Owen Doody, Margaret Graham, Louise Barry, Michelle Kiely, Jonathon O'Keeffe, Jan Dewing, Deirdre Lang, Alice Coffey

**Affiliations:** ^1^ Department of Nursing & Midwifery University of Limerick Limerick Ireland; ^2^ St. Patrick's Hospital Waterford Ireland; ^3^ St. Vincent's University Hospital Dublin Ireland; ^4^ Queen Margaret University Edinburgh Scotland; ^5^ HSE Clinical Strategy and Programmes Division and the Royal College of Physicians of Ireland Dublin Ireland

**Keywords:** older people, older person, person‐centred care, pilot, residential setting

## Abstract

**Background:**

A lack of standardisation of documentation accompanying older people when transferring from residential to acute care is common and this may result in gaps in information and in care for older people. In Ireland, this lack of standardisation prompted the development of an evidence based national transfer document.

**Objectives:**

To pilot a new national transfer document for use when transferring older people from residential to acute care and obtain the perceptions of its use from staff in residential and acute care settings.

**Methods:**

This was a pre‐ and post‐study design using purposive sampling following the STROBE guidelines. The pilot was conducted in 26 sites providing residential care and three university hospitals providing acute care. Pre‐pilot questionnaires focused on current documentation and were distributed to staff in residential care (*n* = 875). A pilot of the new paper‐based transfer document was then conducted over three months and post‐pilot questionnaires distributed to staff from both residential and acute care settings (*n* = 1085). The findings of the pilot study were discussed with multidisciplinary expert advisory and stakeholder groups who recommended some revisions. This consensus informed the development of the final design of the new revised transfer document.

**Results:**

Pre‐pilot: 23% response rate; 83% (*n* = 168) participants agreed/strongly agreed that existing documentation was straightforward to complete but could be more person‐centred. Post‐pilot: 11% response rate; 75% (*n* = 93) of participants agreed/strongly agreed that the new transfer document promoted person‐centred care but recommended revisions to the new document regarding layout and time to complete.

**Conclusions:**

This study highlighted some of the challenges of providing safe, effective and relevant transfer information that is feasible and usable in everyday practice.

**Implications for practice:**

Standardisation and being person‐centred are important determining factors in the provision of relevant up to date information on the resident being transferred.

**What does this research add to existing knowledge about gerontology?**

- This research identifies the components of transfer documentation necessary for safe and effective transfer of older people from residential to acute care.
- The results highlight the importance of balancing the need for person‐centred documentation and pertinent medical information when older people transfer between residential and acute care settings.

**What are the implications for this new knowledge for nursing care with older people?**

- Having person‐centred holistic information about older people and their care needs will improve communication and encourage safer and better patient care on transfer.
- Transfer documentation needs to be comprehensive but easy to use and preferably electronic to reduce errors, particularly in cases of emergency transfers.

**How could the findings be used to influence policy or practice or research or education?**

- This study has highlighted the value of involving all stakeholders including older people themselves in the design and development of a person‐centred and effective transfer documentation.
- The results demonstrate the importance of staff acceptability, ease of use and availability in electronic format, to implement this documentation at national level.

## 1 INTRODUCTION

Internationally, older people account for a high proportion of transfers to emergency departments and admissions to acute hospitals (Barbadoro et al., 2015; Franchi et al., 2017). Older people are the largest group presenting with illness to Irish acute services, accounting for one‐fifth of all emergency department admissions (Department of Health, 2018). Older people transferred from residential care to acute services are accompanied by a transfer document outlining their care needs. However, transfer document information is not always standardised across healthcare settings. International evidence highlights that standardised documentation improves communication between staff by recording important clinical and personal information (Morphet et al., 2014; Tsai & Tsai, 2018).

Recognising the importance of using a standardised document to improve communication, the National Clinical Programme for Older People, supported by Office of Nursing and Midwifery Services Director, Health Services Executive, Ireland, commissioned a project to develop a person‐centred national transfer document for use when an older person is being transferred from residential to acute care settings. This paper reports on the piloting of the document and presents the findings of the pre‐study (existing transfer document) and post‐study (pilot of a newly developed standardised national transfer document).

### 1.1 Background

International evidence highlights elements that should be included in transfer documentation. This includes medical information, vital signs and medications (Cwinn et al., 2009; McCloskey, 2011; Zamora et al., 2012), and information on the older person's comprehensive needs (Campbell et al., 2017; Matic et al., 2011). However, there is a dearth of evidence on what constitutes person‐centred information within transfer documents (Boltz et al., 2013) and research is needed to determine essential components of transfer documentation for effective and safe transfer of older persons (LaMantia et al., 2010).

The funded project aimed to improve the quality and standardisation of transfer documentation for the older person between a residential and an acute care setting (Coffey et al., 2019). Informed by evidence from a literature review, a qualitative study with stakeholders (O'Reilly et al., 2019), consultation with a multidisciplinary expert advisory group^1^ (advisory group) and an expert in person‐centred care, the components and format of a transfer document were identified and developed. It consisted of two sections: one contained biographical and essential medical information, and the other profiled the person's personal preferences and usual health status. As the proposed transfer document would be used nationally, it was agreed to pilot it across several institutions to identify any areas that needed revision before it was put into general use.

This paper presents the results of the pre‐ and post‐study, which explored participants’ perceptions of the design, layout and usability of the pilot, transfer document, as well as compared it with existing transfer documentation. These findings together with advisory group and post‐pilot stakeholder group^2^ consultations were used to further revise and refine the design of the eventual national transfer document. Table 1 outlines the steps in the development of national transfer document.

**TABLE 1:** Steps in the development of a national transfer document

1	Literature review
2	Qualitative study with key stakeholders
3	Consultation with multidisciplinary expert advisory group and expert in person‐centred care
4	Development of transfer document
5	Pre‐ and post‐study of piloting of transfer document
6	Review of findings and consultation by multidisciplinary expert advisory group, research team and stakeholder group
7	Consensus and finalisation of national transfer document

## 2 METHODS

### 2.1 Design

This study was a pre–post survey design, using a questionnaire with a purposive sampling strategy. The aim was twofold. In the pre‐pilot survey, participants in residential care settings were asked to give their views on their existing transfer documentation. In the post‐pilot survey, participants in both residential and acute care settings were asked to give their views on the new transfer document. Participating sites were provided with an onsite study information session and introduction to the pilot transfer document. An explanatory pack (copy of presentation, example of a completed transfer, new transfer document and guidance document) was provided.

Computer‐based documentation is advocated as a way of decreasing time spent on paperwork (Yu et al., 2006) and facilitating multidisciplinary access to accurate and comprehensive information across a variety of care settings (Devriendt et al., 2013). However, paper transfer documentation was used in this study as it was anticipated that changes to the documentation would be required once the data were analysed and electronic formats would not be accessible to all sites at the time of the pilot. This study is reported in line with reports of cohort studies; the Strengthening the Reporting of Observational Studies in Epidemiology (STROBE) (Equator Network, 2019).

### 2.2 Participants

A purposive sample of 26 residential care sites and acute care sites (in three university hospitals) in Ireland's Midwest, South and East regions were recruited. Participants in the residential care setting comprised nurses and health care staff, and participants in the acute care settings comprised nursing, medical, health care and allied health professionals. Participant cohorts were reflective of the staff mix of both settings. Participation included the completion of a questionnaire on their current documentation (residential care settings), agreement to use the new documentation in paper format for a period of 3 months (residential care settings) and completion of a post‐pilot survey (both residential care and acute care settings).

### 2.3 Data collection instruments and method

Each participating residential care site completed a pre‐pilot site profile detailing resident and staff numbers. Pre‐ and post‐pilot survey questionnaires (residential care and acute care services) contained staff demographic questions including category of staff, area currently working and years working in the area. Questions related to staff perception of the current (pre) and new (post) transfer documentation were rated on a 5‐point Likert scale from 1 = strongly disagree to 5 = strongly agree. These questions asked about: layout, ease of use, clarity of information, inclusion of relevant resident clinical and personal information, and whether a multidisciplinary team could use it. Participants were invited to provide overall comments in three open text boxes regarding their thoughts on the transfer document, specific areas of concern and suggestions for improvement.

Pre‐pilot questionnaires were distributed in January 2019. Participants returned questionnaires into a sealed designated collection box within each site. Piloting of the new documentation was conducted over a 3‐month period from February to May 2019. Post‐pilot questionnaires were distributed in May 2019. Questionnaires were returned either to the sealed designated collection box or via post (stamped addressed envelopes provided). Two methods of distribution were used due to time constraints of researchers. Before distribution, all questionnaires were coded and anonymised, codes were assigned to each site.

### 2.4 Ethical considerations

Ethical approval was obtained from the Research Ethics Committees of the three University Hospitals and University leading the research study. The study information and purpose were provided in written participant information sheets. Residential care and hospital site staff were provided with onsite information, explanation and queries answered by researchers. Participant consent was implied by return of the questionnaire(s), and participants were informed that they could exit the study at any stage.

### 2.5 Data analysis

Descriptive statistical analysis was conducted using the SPSS Statistics version 25 for Windows (IBM Corp, 2017). The data from the open‐ended text boxes within the questionnaires were thematically analysed using the Braun and Clarke (2006) six‐phase framework. However, in order to report specifically on participants’ responses to the three topics in the open‐ended questions, the data were subsequently analysed for content. The purpose of content analysis ‘is to organize and elicit meaning from the data collected and to draw realistic conclusions from it’ (Bengtsson (2016):8). Content analysis facilitated direct comparison of pre‐ and post‐pilots of participants’ thoughts, concerns and suggestions for improvement. The data were independently reviewed by two researchers, and initial codes were identified and categorised according to the topic question. Subsequently, the data and initial codes were further analysed, reviewed and agreed by three researchers. The codes for each of the open‐ended topic questions are presented in Table 2.

**TABLE 2:** Themes and codes

Theme	Codes	
Pre‐pilot		
Thoughts on the document	Allergies highlighted Clinical information present Comprehensive Easy to read Easy to complete Long winded Quick to fill in Relevant information on the person	Too detailed Too general Too long Information appropriate for planned hospitalisation Persons care needs are identified Person‐centred
Concerns	Confusing layout Missing relevant information Mobility score absent Not enough spacing Suitability in emergency when have little time Suitability when person is unwell need to give them time Time‐consuming Difficult to include the person Not read by acute staff Relevance of a lot of the information in an acute situation	Repetition Too much information required Complicated so takes too long Don't get to complete it all Don't get to clearly highlight necessary information Takes too long Often, there is a need for follow‐up and clarity Poor compliance in completing so not all information communicated
Suggestions for improvement	Add vital signs, Needs to be shorter Needs to be more user‐friendly Needs more specific information User of colour to highlight Communicate the person's meds Medication history can be presented for follow‐up/clarity Necessary information	Less crowded layout Encourage patient care and focus Non‐acute information can be pre‐populated and include the person Short form can be done with long version to follow Needs to be electronic More space for clinical summaries
Post‐pilot		
Thoughts on the document	All clinical info provided All relevant information provided Comprehensive Easy to complete Easy to fill in, Broadens focus beyond acute problem In‐depth patient care needs provided Info relevant to person‐centred care No need to ring nursing home for information anymore	Standardisation is good Useful and good tool More personal information provided Person‐centred Supports the person Accuracy of information in emergency situations Essential information transferred Can use for patient discharge also
Concerns	First page very busy Irrelevant information asked Lack of space on document Repetition Suitability for acute transfer—don't have the time Too detailed take time to complete Time‐consuming to complete Handwriting can be difficult to read	Need to consult several files to complete Too long Not always valued Poor compliance in completing Ring to get information Tend to ring rather than read Incomplete at times Information missing/not entered Not read
Suggestions for improvement	Needs to be highlighted in colour Info more clearly highlighted Layout could improve More info required Pre‐filled sections and baselines Patient input and participation Add reason for transfer to first page Colour‐coded system Condense and be more concise DNR/CPR first page	Mobility score to be recorded Need to contain resuscitation information Needs to be shorter Not to use paper‐based version More concise Summary sheet of patient ADLs Education of acute staff on relevance Electronic version Formatting and layout

## 3 FINDINGS

### 3.1 Pre‐pilot results

Twenty‐six residential settings agreed to participate in the study and returned questionnaires. Pre‐pilot questionnaires sought the perceptions of residential staff (nursing and health care) (*n* = 875) on the transfer documentation currently in use. There was a 23% response rate (*n* = 202) specifically comprising 47(23%) (Midwest), 68 (33%) (South) and 87 (44%) (East) regions.

### 3.2 Site and participant demographics

The majority (*n* = 137, 68%) were nurses providing direct care. The remaining 32% (n = 65) comprised clinical nurse managers, clinical nurse specialists, directors of nursing/person in charge, and healthcare assistant/health manager/student nurse. The highest level of education among staff was a bachelor's degree (48%, *n* = 96). Table 3 provides an overview of pre‐pilot participants.

**TABLE 3:** Overview of pre‐pilot participants

Category of participant	Number	Percentage
Nurse	137	68%
Clinical nurse manager	44	22%
Clinical nurse specialist	2	1%
Director of nursing/person in charge	18	8%
Other (student nurse, healthcare assistant, senior health manager)	1	1%

### 3.3 Current transfer document components

**FIGURE 1:**
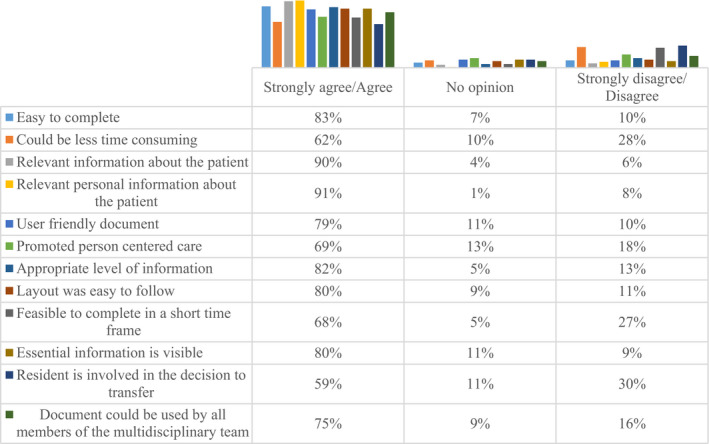
Current transfer document components

Participants expressed their views about their current transfer document on a scale of strongly agree, agree, no opinion, disagree and strongly disagree. Figure 1 contains an overview of participants’ perceptions of the current transfer document.

Participant perceptions were positive in all areas and very positive in relation to ease of completion (83%), user‐friendliness (79%), layout easy to follow (80%) and relevance of information about the patient (90%). However, there was less agreement that the current documentation promoted person‐centred care (69%) and least agreement (59%) with the statement regarding resident involvement in the decision to transfer. It is worth noting that most sites used documentation in electronic format. Open‐ended questions on the pre‐pilot questionnaire provided some insights into participants’ perceptions of what needed to improve.

### 3.4 Open‐ended text results

Three open‐ended questions were asked: ‘In general what are your thoughts on the Transfer Document’, ‘Do you have any specific areas of concern about the documentation’ and ‘Do you have suggestions for improvement’. 293 comments were provided (60.9% of total). Themes and codes are outlined in Table 2. Participants commented that information was identified as relevant and person‐centred but was time‐consuming and not always read by acute care staff. Electronic documentation was advocated for.

### 3.5 Thoughts

Participants’ thoughts on the current transfer document included their views on the length of the document and time required to complete, level of detail and relevancy of information and the person‐centredness of the document. Many participants believed that in the current document, ‘*information recorded about the patient was relevant’ (S18)*.

Participants also perceived that their current transfer document was person‐centred. They could pre‐populate areas in consultation with the resident about their personal needs and clinical needs.

It is a very person‐centred document and give a holistic assessment of the resident being transferred to the acute sector (S3)

Additionally, participants identified that sufficient time is required to highlight essential clinical and personal information.

### 3.6 Concerns

Participants raised concerns about current transfer documents relating to clarity, detail, repetition, missing relevant information, incompletion and the length of time it took to complete. This was of particular concern in emergency situations when a patient needed to be transferred quickly.

I think it is too long and difficult to complete in an emergency it's not feasible, I have only filled it out completely twice only because we had time while we waited for an ambulance but you don't get the time when an ambulance is coming in a few minutes (S15)

Participants were also concerned that transfer document was not read by the acute staff and that although they currently provided what they considered to be adequate transfer information, they were often contacted by acute care staff for follow‐up information about the resident. This raises questions about how and what information is communicated on the document and existing communication practices between sites.

Most times, we had filled in more than enough into our own transfer letter, A&E would still ring us and ask about the information that was written on the transfer letter (S17)

### 3.7 Suggestions for improvement

Participants had a number of suggestions for improvement including having essential information with pre‐population of non‐acute information, having a more user‐friendly design and being electronic.

Participants identified the challenge of balancing the requirements of providing essential information (clinical and personal) with wanting a form which is short, quick and easy to complete.

A transfer letter should be a document that contains all the relevant information of a resident which helps to commence patient‐centred care in a new setting, it should be easy to complete but not too long (S15)

The layout of the document was thought by participants to have a direct impact on whether it was completed correctly. Furthermore, it was clear that some residential care sites already used electronic documents and staff in these sites were reluctant to return to a paper‐based system, indicating an area of improvement.

It (a new document) could be uploaded to (software name) and have most of the sections pre‐populated (S20)

### 3.8 Post‐pilot results

Nineteen residential sites and three acute care sites agreed to participate in the post‐pilot survey (*n* = 1085). Seven of the 26 pre‐pilot residential care sites declined to participate in the post‐pilot survey due to time constraints. Although reminders were sent, only 124 completed questionnaires were returned, resulting in a response rate of 11% comprising 34 (27.4%) (Midwest), 40 (32%) (South) and 50 (40%) (East) regions.

### 3.9 Site and participant demographics

Fifty per cent (*n* = 62) of participants were nurses who provided direct care, 29% (*n* = 37) were clinical nurse managers and advanced nurse practitioners; 10% (*n* = 12) were allied and medical professionals, 6% (*n* = 7) were persons in charge; and 5% (*n* = 6) were student nurse/healthcare assistants/health manager. The highest level of education among staff was a bachelor's degree (40%, *n* = 50). Table 4 provides an overview of post‐pilot participants.

**TABLE 4:** Overview of post‐pilot participants

Category of participant	Number	Percentage
Nurse	62	50%
Clinical nurse manager	34	27%
Advanced nurse practitioner (cANP/ANP)	3	2%
Director of nursing/person in charge	7	6%
Doctor (NCHD or consultant)	7	6%
Physiotherapist	3	2%
Occupational therapist	1	1%
Pharmacist	1	1%
Other (student nurse, healthcare assistant, senior health manager)	6	5%

### 3.10 Pilot transfer document components

**FIGURE 2:**
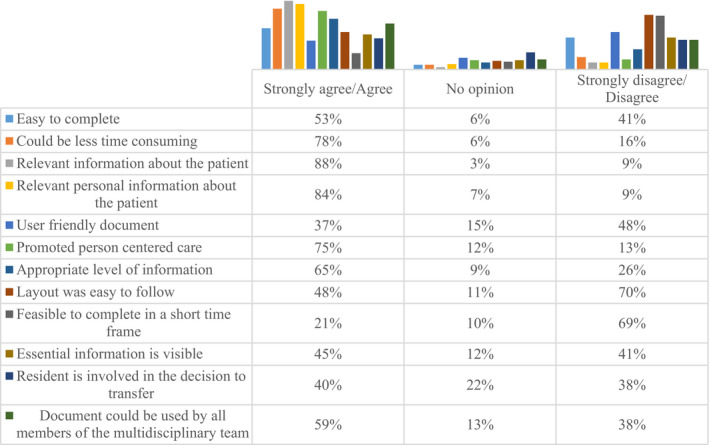
Pilot transfer document components

Participants expressed their views on a scale of strongly agree, agree, no opinion, disagree and strongly disagree. Figure 2 contains an overview of participants’ views. When comparing pre‐ and post‐pilot responses, it is evident that participants had reservations about the new document (Figures 1 and 2). The scores in the post‐pilot survey with the exception of person‐centred care were lower than current documentation. Although most respondents (88%) strongly agreed/ agreed that relevant information was provided about the patient and a higher percentage of participants post‐pilot (75%) perceived that the pilot document promoted person‐centeredness in comparison with their current documentation (69%), lower scores related to user‐friendliness (37%), layout easy to follow (48%) and feasibility to complete in a short time (21%).

### 3.11 Open‐ended text results

In the post‐pilot survey, three open‐ended questions were asked: ‘In general what are your thoughts on the National Transfer Document’, ‘Do you have any specific areas of concern about the documentation’ and ‘Do you have suggestions for improvement’. The themes and codes are outlined in Table 2, and 188 comments were provided (39.09% of total). The participants commented that the pilot document was useful and comprehensive and promoted more person‐centred care. The layout and length of the document and therefore time to complete in practice were problematic, along with the fact that the document was in paper format.

### 3.12 Thoughts

Participants’ thoughts on the pilot transfer document centred on comprehensiveness, relevancy, clarity, person‐centredness, length of the document, ease of completion and time required to complete. In general, they reported that the document was easy to complete, comprehensive and useful.

Easy and clear to follow (S22)

very useful (S29)

Participants felt having a standardised document was good as long it was completed. Participants from both sites felt the transfer document would improve communication both between and within care settings. Many participants from acute care noted that due to the comprehensive nature of the information, there was less of a need to follow up with residential care sites. However, if the document was not completed fully or not read, phone calls were still made.

The document has more information and detail is provided on it…now there is no need to ring nursing home for information anymore (S20)

Participants also endorsed the person‐centred nature of the document and identified its focus beyond acute problems to including personal aspects and care needs of the individual. Identified as being key to being person‐centred was the involvement of the person, and it was suggested this aspect of the document could be completed when the resident is not unwell.

All patient care needs, clinical and personal are recorded (S29)

Encourages person‐centred care (S15)

It encourages hospital members to not only treat the acute problem but to help residents to return to their baseline and to understand how the acute illness in fact has changed the patients' baseline/overall condition (S15)

### 3.13 Concerns

Participants voiced several concerns about the pilot transfer document, and these related specifically to lack of specific information, design, poor compliance and time. Most participants stated that completing the document was very time‐consuming. This was anticipated as a problem if the resident required an emergency transfer. However, participants agreed it could be beneficial if correctly completed.

Time consuming but could be excellent if filled out properly (S3)

Too time consuming; one would have to start completing it and then call the ambulance, just to make sure it is accurate and whole (S15)

Some participants were concerned with the prioritisation and/or omission of certain information. They also felt that due to design and layout, that important information could be lost or not emphasised enough.

Relevant and essential info such as resuscitation wishes are not contained and the layout is packed and essentials not stressed enough or visible (S17)

Some participants did think positively about the document but had concerns about whether it would be completed properly.

Should work when filled out correctly and used efficiently (S29)

### 3.14 Suggestions for improvement

Participants proposed changes to the pilot document such as relooking at layout, being concise, adding more information, patient involvement in completing document and computerisation. There was a resounding call for a computerised rather than paper‐based version. Staff argued that computerising this document would reduce errors due to illegible handwriting, less time would be spent writing down information, and it would enable the document to be sent swiftly in emergency situations.

It would be easier and quicker to use if the document was computerised and handwriting can be difficult to read and takes longer (S23)

Many participants wanted a one‐page summary document with person‐centred information to accompany an existing transfer document. It was suggested that the summary document could be populated in advance within the residential care service.

Maybe a summary, person‐centred sheet that is pre‐filled (S11)

Changes in the layout were suggested for effective use. These included making the document shorter, adding more space for certain sections and the reason for transfer.

Front pages should have the emergency details of condition and current treatments being undertaken (S9)

Shorten the form and include only relevant information regarding the reason for transfer (S22)

### 3.15 Review of the findings

The study's results were presented to the advisory group. Revisions to the document were undertaken to address content, length, spacing, visibility and layout. Two stakeholder consultation panels were convened to review and discuss these proposed amendments with both the advisory group and the research team. This stakeholder group comprised participants from the pilot study residential and acute care sites and service user advocate representative. Consensus on the design was reached and agreed that the revised transfer document would be entitled the ‘National Transfer Document and Health Profile’. It consists of a transfer information section and a health profile section. The transfer information section includes demographic and medical information pertinent to care in the acute setting. The health profile section contains individualised person‐centred information and is completed with the resident and may be pre‐populated. It is to be sent with the transfer information when the resident is transferred (Coffey et al., 2019) (See Appendix S1).

## 4 DISCUSSION

This study aimed to identify residential care participants’ (nurses and health care assistants) perceptions of their current resident transfer document (pre‐pilot) and to identify participants’ (residential care and acute care staff) perceptions of the feasibility and usability of the pilot transfer document and its applicability to the care facility. Definitions of pilot studies include focusing on acceptability and feasibility issues of a tool being piloted (Spurlock, 2018). The findings proved invaluable in determining the properties of a transfer document acceptable to users. This study highlights the importance of developing a transfer document, which is both ‘user‐friendly and comprehensive’. Similar to findings by O'Reilly et al., (2019), participants of this pilot study were interested in using a transfer document, which provides clear, concise and person‐centred information. The findings emphasised the need for a transfer document, which simultaneously identifies the patient's specific nursing and medical needs and provides person‐centred knowledge and understanding of the patient's specific individual needs. Furthermore, the transfer document must meet the criteria of being user‐friendly in language, design and layout, be easily and quickly completed, but at the same time be comprehensive in content about the patient's particular care needs. These requirements presented a challenge in how to best change and amend the national transfer document to suit the needs of both patient and service providers.

While participants’ perceptions of their current documentation indicated that they were generally satisfied, in that it was familiar and easy to complete, they also acknowledged that there were difficulties such as lacking a level of person‐centeredness. Person‐centred holistic care is a cornerstone of gerontological nursing (McCormack & McCance, 2017) and a core standard of practice for nurses working with older people (Nursing & Midwifery Board of Ireland, 2015). Similar to previous studies (Dizon et al., 2017), the findings indicate that deficits in the current documentation often led to follow‐up calls from acute care services to clarify information and/or seek missing information. Frequent calls are time‐consuming, add to the burden of work and may have an adverse impact on efficiency and effectiveness. Interestingly, participants perceived that this type of additional communication between settings was reduced in the post‐pilot period. Resident transfers should be seamless for patients and carers, this can only occur through better coordination and communication (Shaw et al., 2011) where unambiguous language and clear, comprehensive communication are provided regarding patient care (De Groot et al., 2019).

The findings of this study reinforce the literature emphasising that a standardised and consistent layout is an important determining factor in the provision of relevant up‐to‐date information on the resident being transferred (Arendts et al., 2013; McCloskey, 2011; Robinson et al., 2012). The use of a standardised national transfer document is important in providing coordination and continuity of health care for older people being transferred, especially as older people often have comorbidities requiring complex care management.

It is acknowledged that there were differences in the pre‐ and post‐pilot groups, in that the pre‐pilot sample was drawn from residential care sites only and comprised many nurses, whereas the post‐pilot sample comprised both residential care participants (mainly nurses) and acute care participants, thus accounting for the inclusion of medical doctors and allied healthcare professional in the post‐pilot sample (10% of post survey sample). This different composition of the post‐pilot sample may have had a bearing on the results, and this is addressed in exploring the purpose of the pre‐pilot questionnaire and the different focus of the post‐pilot questionnaire. The lack of medical and allied healthcare professionals from the pre‐pilot survey reflects the staff mix within the residential care setting. In the planning of the study, it was decided that the pre‐pilot sample would focus on the residential care setting only and seek their views on their current documentation and so compare this with the pilot document. Inclusion of the pre‐pilot survey data yielded useful information on what worked well and what did not with current documentation, all of which informed subsequent adaptations to the post‐study refinement and amendment of the national transfer and health profile document. The post‐pilot sample focused on both the views of those completing the transfer document (residential care) and those receiving it (acute care). Therefore, how the pilot document was viewed would have been shaped and influenced by the priorities and perspectives of those using it. The post‐pilot findings provided valuable information on the design, usability and applicability of the transfer document both from the perspectives of those completing the document (residential) and from the perspectives of those receiving and interpreting the document (acute care). Participants were concerned about accuracy of information, of it not being easily visible and about the time required for completion especially in cases of emergency transfers. Accurate and complete documentation is crucial to the delivery of quality health care (Voyer et al., 2014).

Although most transfer documents were reported as the traditional paper and pen version, electronic versions of transfer documents were identified (Campbell et al., 2017). In tune with Yu et al., (2006), participants in this study favoured a computerised document and recommended some pre‐population of data where appropriate, to save time and promote person‐centred care. Like previous research (Murray & Laditka, 2010; Zamora et al., 2012), this study finds that an electronic transfer document is perceived as a means of reducing errors when transferring residents. Furthermore, it would provide comprehensive and accurate information.

This study highlights the need to promote a culture that supports both effective and person‐centred documentation and recognises the importance of allocating time to document. In response to the findings of this study, amendments and changes to the document were made as outlined previously.

### 4.1 Limitations and recommendations

The sites in this study represented a good geographical spread of rural and urban areas although located within one county. While the pilot sample provided a valuable perspective on person‐centred transfer, it is acknowledged that older people and their families are absent at this stage of the research project. A limitation of this study was the low response rate for post‐pilot questionnaires (11%). It is unclear, and there is no evidence to suggest that this may be due to high workloads and limited time among staff or lack of awareness of study. The results of the pilot provided valuable information to inform the development of the final document. The views of various health and social care staff were represented in the pilot and in the final post‐pilot consultation along with key stakeholders involved in the delivery, planning and design of services for older people. The older person's perspective was included in the final consultation process through the involvement of advocates. The final document will now be available electronically, a welcomed support from governing health bodies, thereby increasing accessibility and accessibility. Future research to implement the transfer document will use a public and patient involvement (PPI) approach, which will enable older people to become involved with research within residential and acute care settings.

## 5 CONCLUSION

This paper describes the results of a pilot study that combined with a consultative process resulted in an evidence‐based consensus document that may provide relevant and appropriate person‐centred information on transfer between residential and acute care. Incorporating a consultative process has the potential to develop user‐friendly and comprehensive documents. The methodology used facilitated the inclusion of all stakeholders. Employing an electronic document offers quick and efficient access to patient's details and valuable information.

## CONFLICT OF INTEREST

No conflict of interest has been declared with the authors.

## AUTHOR CONTRIBUTIONS

AC, DT, AF, JO'D, PM, PO'R, BO'B, JM, OD, MG, LB, MK and JO'K made substantial contributions to concept and design, acquisition of data, or analysis and interpretation of data. AC, DT, AF, JO'D, PM, PO'R, BO'B, JM, OD, MG, MK, JO'K, JD and DL helped to draft the article or revising it critically to important intellectual content. AC, DT, AF, JO'D, PM, PO'R, BO'B, JM, OD, MG, LB, MK, JO'K, JD and DL gave final approval of the version to be published and each author should have participated sufficiently in the work to take public responsibility for appropriate portion of the context. AC agreed to be accountable for all aspects of the work in ensuring that questions related to the accuracy or integrity of any part of the work are appropriately investigated and resolved.

## DISCLOSURE

The final article version has been agreed by all authors. The criteria as per the International Committee of Medical Journal Editors (ICMJE) have been upheld by all authors.

## Supporting information

App S1

## Data Availability

Data are available from the authors with the permission of the Health Service Executive.

## Footnotes

^1^
Multidisciplinary expert advisory group: representatives included the following: service users, nurses and allied healthcare professionals working with older people in acute, primary, community and continuing care settings, providers of nurse education and practice development, and regulatory, advocacy and policy representatives

^2^
Stakeholder group comprised of nurses, social care professional and service user representative
