# Supplementary material for: Towards the development of a national patient transfer document between residential and acute care—A pilot study
Source: Int J Older People Nurs. 2021 Mar 24;16(4):e12374. doi: 10.1111/opn.12374 (PMC8365739; doi:10.1111/opn.12374)
Supplement: Supplementary file 1 — App S1 [file OPN-16-e12374-s001.docx]

**Transfer Document and Health Profile**

**NATIONAL TRANSFER DOCUMENT AND HEALTH PROFILE FOR RESIDENTIAL CARE FACILITIES**

*The Health Profile, up to date copy of Medication List and Administration Record of the person being transferred, should accompany this document. Please ensure that these and any other relevant information is attached.*

| **Priority 1: THINGS THAT YOU MUST KNOW ABOUT ME**  This Section should be completed at time of transfer | | | | | | | | | |
| --- | --- | --- | --- | --- | --- | --- | --- | --- | --- |
| **ISBAR**_3_  **Communication Framework** (*Based on NCG No.11, NCEC, DOH, 2015 and NMPD DML, 2018) | | | | | | | | | |
| **I** | **Identify:** Identify yourself, who you are talking to and who you are talking about | | | | | | | | |
|  | Recipient of Information: | | | | | | | | |
|  | GP Name: | | | GP Number: | | | | | |
|  | Referred by:  e.g. GP, GP Out of Hours, Nurse in charge | | | Seen by GP *(Please circle)*: Y/N | | | | | |
|  | At present the resident is receiving care in: | | | | | | | | |
|  | Unit Name : | | | Unit Telephone Number: | | | | | |
|  | Health Mail Address­­­­­ of Unit/ Email Address of Unit: | | | | | | | | |
|  | Nurse in Charge of Unit: | | | Key worker *(If applicable):* | | | | | |
|  | Named Designated Representative/ Contact Person (including wards of court): | | | | | | | | |
|  | Designated Rep/Contact Person notified of transfer *(Please circle)*: Y/N | | | Phone Number: | | | | | |
|  | Medical Card *(Please circle):* Y/N | | | Health Insurance *(Please circle)*: Y/N | | | | | |
|  | Religion / Spiritual Needs: | | | Ethnicity: | | | | | |
| S | **Situation:** What is the current situation/change in condition, concern, observations etc?  Why am I (resident) being transferred? | | | | | | | | |
|  | Brief summary of resident's current status/identification of the problem requiring transfer. | | | | | | | | |
| B | **Background**: Summary of Treatment to Date, Relevant Medical/Surgical History, Vital Signs (Please complete with resident if possible) | | | | | | | | |
|  |  | | | | | | | | |
|  | **MY MEDICAL INFORMATION** | | | | | | | | |
|  | Have I (resident) been involved in the decision to transfer me to hospital? (*Please circle):* Y/N  If no, please state reason: | | | | | | | | |
|  | **A copy of my medicines prescription is attached** *(Please circle):* Y/N | | | | | | | | |
|  | Do I present as disorientated *(Please circle):* Y/N | | | | Do I present with symptoms of pain*:* Y/N | | | | |
|  | Do I present with a choking risk (*Please circle*): Y/N  *(Please see eating and drinking in my health profile)* | | | | | | | | |
|  | My bowels last opened Time__:__ Date __/___/___  I last passed urine Time__:___ Date ___/__/___ | | | | | | | | |
|  | I use breathing support (*Please circle as appropriate*): BiPap NIV LTOT  Please provide details: | | | | | | | | |
|  | I have a history of adverse drug reactions/ allergies *(Please circle):*  Y/N  If Yes please specify: | | | | | | | | |
|  | I have a history of adverse other reactions/ allergies *(Please circle):*  Y/N    e.g. (anaphylaxis, medication allergy, food allergies and/ or intolerances *etc.*) *(Please give details including what my reactions would be)* | | | | | | | | |
|  | I have an Advanced Health Care Plan/ Directive *(Please circle):* Y/N/ copy attached  I have an End of life care plan dated and attached: *(Please circle):* Y/N  Active Safeguarding Concerns *(Please circle):* Y/N  (*If Yes Please Contact Residential Care Setting*) | | | | | | | | |
|  | I **currently** have a health-care associated infection *(Please circle):* Y/N/Unknown  *If known* *please circle* : HCAI/ MDRO/ BBV^^[[1]](#footnote-1)^^ status, Influenza, Norovirus, Hep B, Hep C, HIV, *Clostridium difficile*, MRSA, CPE/ CPE contact, VRE  **Other** *(Please specify):* | | | | I have **a history** of a health-care associated infection status *(Please circle):* Y/N/Unknown  *If known please circle* : HCAI / MDRO/ BBV^^[[2]](#footnote-2)^^ status, Influenza, Norovirus, Hep B, Hep C, HIV, *Clostridium difficile*, MRSA, CPE, VRE  **Other** *(Please specify):* | | | | |
|  | **I have been informed of my HCAI/ MDRO/ BBV status** (*Please circle):* Y/N | | | | **Eradication / screening protocol attached** (If relevant)*(Please circle):* Y/N | | | | |
|  | **SUMMARY OF TREATMENT TO-DATE** | | | | | | | | |
|  |  | | | | | | | | |
|  | **RELEVANT MEDICAL/SURGICAL HISTORY/KEY MEDICAL INFORMATION** | | | | | | | | |
|  |  | | | | | | | | |
|  | **VITAL SIGNS** | | | | | | | | |
|  | **Recorded by:** | | | | **Time recorded:** | | | | |
|  | **B.P.** | **Pulse Rhythm**  Regular / Irregular | | | **Pulse Rate** | **Temperature** | | **Respiratory Rate:** | |
|  | **O_2_ Sat (R/A):** | **O_2_ Sat**  (O_2_ therapy): | | | **Blood Sugar:** | **GCS:** | | **AVPU** | **Other:** |
| **A** | **Assessment: What is the problem/your assessment of the situation?** | | | | | | | | |
|  |  | | | | | | | | |
| **R** | **Recommendation, Read-back, Risk** | | | | | | | | |
|  | Specify your (nurse) clinical recommendations | | | | | | | | |
|  | Identify possible risks | | | | | | | | |
|  | Date and time: | | Signature: | | | | Print Name | | |

| Checklist of Supporting Documentation ***Please attach Health Profile** |
| --- |
| Health Profile Y/N  Medication List Y/N  Medication Administration Record Y/N  End of Life Care Plan (If applicable) Y/N  Advance Care Plan/Directive *(If applicable)* Y/N  Medical Transfer Letter Y/N  Enteral Feeding Regimen *(If applicable)* Y/N  Healthcare Associated Infection Protocol Y/N  List accompanying equipment :  Other relevant information: Please state: |

| MY HEALTH PROFILE  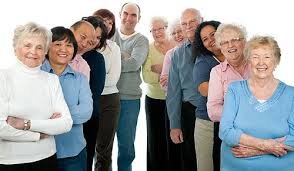  **THINGS THAT WORRY OR UPSET ME:**  **HOW I RESPOND TO NEW ENVIRONMENTS OR EVENTS:**  **SPECIAL ITEMS I LIKE TO HAVE WITH ME**  ***(If applicable):***  **I CURRENTLY RESIDE AT** (Nursing Home/Community Hospital)  **MY NAME IS**:  **I WANT TO BE CALLED**:  **MY DATE OF BIRTH** __ /__/____  **WHAT I WANT YOU TO KNOW ABOUT MY IDENTITY** (e.g. Gender)  **THINGS I LIKE:** (e.g. what makes me happy, things I like to do, see or talk about)  **IMPORTANT PEOPLE IN MY LIFE**:  **WHEN I SEEK ADVICE, I ASK THE FOLLOWING PEOPLE:**  **My Personal Preferences**      **My Personal Preferences**  **SMOKING**  **I smoke.** *(Please circle):* Y/N  **If yes, how much daily?**  **I vape** *(Please circle):* Y/N  **ALCOHOL USE**  **I drink alcohol** *(Please circle):* Y/N  **If yes, how often and how much?**  **MENTAL HEALTH**  **How do I describe my mental well-being?**  **MY SLEEP PATTERN**  **Usual bedtime:**  **Usual clothing:**  **Time of settling:**  **Time of waking:**  **You can help me settle by:**  **I usually sleep in bed** *(Please circle):* Y/N  **I usually have ____ pillows**  **I usually call out for assistance** *(Please circle):* Y/N  **I need a bell/I need an adapted bell** *(Please circle):* Y/N      **HOW I AM USUALLY**  ***Please circle answers unless otherwise indicated***  **MY SKIN INTEGRITY**  Intact/ Grade 1/ Grade 2 /Grade 3 /Grade 4  ***(Please complete one scale below as appropriate)***  **Water low Score:** 10+ (at risk), 15+ (high risk), 20+ (very high risk)  **Braden Score:** <11 (high risk), 12-14 (moderate risk), 15-16 (low risk), ≥ 17 (no risk)  **Norton Score:** ____________  **I use a pressure-relieving device: Y/N**  *(Please specify):*  **Wound location:** *(If applicable)­­­­­­­­_____________*  **Dressing used :**(*If applicable*)_______________  **Dressing used** (*If applicable*):  **MY COMMUNICATION**  **My comprehension:**  No Difficulty/ Mild / Moderate/ Severe  **My expression:**  No Difficulty/ Mild / Moderate/ Severe  **My first language is: ____________________**  **I need an interpreter:** Y/N  **How to support me to communicate e.g. key phrases / terms for understanding/ communication board:**  **SIGHT: I wear glasses**: Y/N  **Other**____________  **HEARING: I wear hearing aids**: Y/N  **What aids are with me:**  Spectacles/ Hearing aids/Dentures/ Assistive Technology/Communication boards  **I need a bell/I need an adapted bell** *(Please circle):* Y/N  **MY MOBILITY**  Independent / Supervision  Assistance x1/ Assistance x2 Immobile/Wheelchair  Standing Hoist /Full Hoist  **I have a mobility aid: Y / N**  Walking Stick/ Frame/ 4 Wheeled Walker/  3 Wheeled Walker /Wheelchair  **I am at risk of falls: Y/N**  **My Functional Level:** (Barthel /20)  **Clinical Frailty Score:**  *(Specify scale used)*  **Other**____________      **HOW I AM USUALLY**  ***Please circle answers unless otherwise indicated***  **MY WEIGHT_____________**  **Date recorded**: __/___ /___  **Any recent change**: Y/ N  **Specify loss/gain:**    **M.U.S.T score**:  **I have a feeding tube in place**: Y/N  **If yes, Please indicate type**   - Nasogastric (NG) - Nasoduodenal (ND) - Nasojejunal (NJ) - Gastric (G) - Gastrojejunal (GJ) - Jejunal (J)     **Size**: ___  **Date** **last inserted**: __ /__ /__    **Regimen attached:** Y/N  **I wear dentures/bridges:** Y/N    **I have crowns/implants:** Y/N  **MY NURTRITION**  **I require assistance with eating/drinking:** Y/N  **Support I may need with eating/drinking** *(please specify)_____________*  **My Foods & Drinks/ Modification Requirements:** *(please circle)*  **Level 0-Drinks** (Thin),  **Level 1-Drinks** (Slightly Thick),  **Level 2-Drinks** (Mildly Thick),  **Level 3-Drinks & Foods** (Moderately Thick-Liquidised),  **Level 4-Drinks & Foods** (Extremely Thick- Pureed),  **Level 5-Foods** (Minced and Moist),  **Level 6-Foods** (Soft and Bite Sized),  **Level 7-Foods** (Easy to Chew/Regular)  **If unsure, please describe:**  **Special diet:** Y/ N *(Please specify­­­­­­­­)*  **Fluid restriction:** Y/N  **ADDITIONAL INFORMATION**  **MY NORMAL BOWEL PATTERN**  I am continent: Y/N  I am not fully continent: Day/Night /N/A  Continence-wear type I use *(If applicable):*  How often I need to go to the toilet:  ___ (hours)  I have a Stoma in place: Y/N  Equipment required:  **MY NORMAL URINARY PATTERN**  I am continent: Y/N  I am not fully continent: Day/Night /N/A  Continence-wear type I use *(If applicable):*  How often I need to go to the toilet:  ___ (hours)  **I have a urinary catheter in situ: Y/N**  **Last changed**: / / **Size:**  **Type:** Urethral or Supra-pubic  I have a stoma in place: Y/N  Equipment required:        **HOW I AM USUALLY**  ***Please circle answers unless otherwise indicated*** |
| --- |

**make your care more individual to your needs**

**Please let us know any further information that would help make your care more individual to your needs**

1. HCAI= Healthcare- associated Infection/ MDRO= Multi- drug Resistant Organism / Blood- Borne Virus= Hepatitis B, Hepatitis C, Human Immunodeficiency Virus [↑](#footnote-ref-1)
2. HCAI= Healthcare- associated Infection/ MDRO= Multi- drug Resistant Organism / Blood- Borne Virus= Hepatitis B, Hepatitis C, Human Immunodeficiency Virus [↑](#footnote-ref-2)
